# Clinical Toxicology of Vitamin D in Pediatrics: A Review and Case Reports

**DOI:** 10.3390/toxics11070642

**Published:** 2023-07-24

**Authors:** Jutti Levita, Gofarana Wilar, Ika Wahyuni, Lidya Cahyo Bawono, Tiara Ramadaini, Rohani Rohani, Ajeng Diantini

**Affiliations:** 1 Department of Pharmacology and Clinical Pharmacy, Faculty of Pharmacy, Padjadjaran University, Sumedang 45363, Indonesia; g.wilar@unpad.ac.id (G.W.); ajeng.diantini@unpad.ac.id (A.D.); 2 Master Program in Pharmacy, Faculty of Pharmacy, Padjadjaran University, Sumedang 45363, Indonesia; ika22005@mail.unpad.ac.id (I.W.); lidya22002@mail.unpad.ac.id (L.C.B.); tiara22013@mail.unpad.ac.id (T.R.); rohani21001@mail.unpad.ac.id (R.R.)

**Keywords:** cholecalciferol, ergocalciferol, hypercalcemia, vitamin toxicity

## Abstract

Intoxication of vitamin D is not a common case in pediatrics. Vitamin D supplements are sold as OTC drugs; however, there is a lack of public education about the permissible limits of vitamin D intake which may lead to vitamin D toxicity (VDT). This review aims to give insights to readers or practitioners about the clinical toxicology of vitamin D in pediatrics, which includes the mechanism of VDT, case reports, and the management of vitamin D poisoning. VDT refers to serum 25(OH)D levels, particularly when the level exceeds 100 ng/mL (250 nmol/L) or is defined as hypervitaminosis D. Hypercalcemia is a common condition of vitamin D toxicity. Vitamin D and its metabolites in moderate levels can induce hypercalcemia, as indicated by the elevation of osteoclastic bone resorption, the presence of calcium in renal tubules, intestinal calcium intake (through increased production of calcium-binding protein in enterocytes), and the decrease of parathyroid hormone synthesis. VDT in pediatrics can be managed by discontinuing vitamin D intake; using activated charcoal, furosemide, prednisone, and calcitonin; rehydration using intravenous sodium chloride 0.9%; and dextrose fluid therapy. It is important for parents to be more careful when providing vitamin D to their children.

## 1. Introduction

Vitamin D (calciferol) is a fat-soluble vitamin which plays many essential roles, including the absorption of calcium and phosphorus for bone and tooth metabolism [1,2,3], while it also regulates immune function [4,5,6,7], reduces diabetes mellitus risk [6,8,9], improves cardiovascular health [6,9], promotes cardiovascular health [6,10,11], prevents the abnormal growth of cancer cells [6], regulates the secretion of certain hormones, differentiation, and proliferation of cells [7], stimulates nitric oxide production [10], protects nerves, reduces migraine attacks [12], and maintains musculoskeletal health [3,7,13]. Vitamin D is classified into two types: vitamin D2 from dietary intake and vitamin D3 from sun exposure [14,15].

Vitamin D is metabolized to its active form, 25-hydroxyvitamin D (25(OH)D), by cytochrome P450 mixed-function oxidases (CYP450s). Vitamin D levels are based on the serum levels of 25(OH)D [13,15,16]. Normal serum 25(OH)D levels are ±30–100 ng/mL [16,17]. A patient is categorized as having a deficiency when the serum 25(OH)D levels are <20 ng/mL and an insufficiency when the serum 25(OH)D levels are 20–29 ng/mL, while toxicity of vitamin D occurs when the serum 25(OH)D levels are >150 ng/mL [15,16]. The dose of vitamin D needed by pediatrics under 12 months is 400 IU/day, whereas babies over 12 months need 600 IU/day [13]. However, long-term therapy of vitamin D may lead to vitamin D toxicity (VDT) or hypervitaminosis D. VDT is a condition where the level of vitamin D or serum 25(OH)D in the body exceeds the normal limit for daily consumption. Acute VDT may occur when the dose reaches 10,000 IU/day and a chronic dose is >4000 IU/day for several weeks [15]. Vitamin D3 supplementation of 700 IU/day for 12 weeks may increase the levels of serum 25(OH)D to >32 ng/mL and improve the health-related quality of life (HRQoL), muscle strengthening, and performance in children [18,19]. A dose of 50,000 IU administered to pediatrics for a duration longer than 9 weeks could increase serum 25(OH)D levels by approximately 27 ng/mL [9]. The recommendation of vitamin D supplementation for pediatrics is summarized in Table 1.

Vitamin D is important for pediatrics to support the growth and development of bones, teeth, body weight, height, and to prevent fractures [18,22]. Children who are deficient in vitamin D have a 2000-fold risk of fracture compared with those with normal vitamin D levels [22]. Therefore, providing the right doses of vitamin D supplements is essential for children and needs to be considered due to their immature digestive systems [15]. Intoxication, particularly excessive doses of vitamin D, may lead to hypercalcemia, hypercalciuria, an imbalance in bone metabolism, fatigue, diarrhea, dehydration, weight loss, kidney stones, confusion, psychosis, nausea, and parathyroid hormone imbalance [1,13]. In conditions of high vitamin D levels, the monitoring of calcium, phosphorus, parathyroid hormone (PTH), and creatinine levels should be carefully performed [23].

Considering the facts that high doses or long-term administration of vitamin D supplements to infants and children may lead to intoxication, this review aims to provide insights to the readers or practitioners about the clinical toxicology of vitamin D in pediatrics, which includes the mechanism of VDT, case reports, and management of vitamin D poisoning. Briefly, articles were initially searched in the PubMed database using the keywords fat-soluble vitamins OR vitamin D, filtered to a publication period during 2013–2023, and limited to publications in free full-text English, with the article types being randomized controlled trial and clinical trial. The collected articles were further meticulously screened by titles and abstracts, and only those studied in children and infants were selected.

## 2. Clinical Features and Adverse Events of Vitamin D

Vitamin D is known to generally have dose-dependent effects [24]. The function of vitamin D is to maintain mineral metabolism, bone health, and muscle function, and to regulate the immune system and respiratory health [25,26,27]. A high vitamin D dose of 7000 IU/day for 12 months elevates neuromuscular motoric skills in children and adults aged 19–25 years with human immunodeficiency virus (HIV) [27]. Vitamin D in infants is effective in improving bone health and preventing rickets at a dose of 400 IU/day [28]. The administration of vitamin D at 400 or 800 IU/day for 4 weeks to premature infants with a gestational age of 24–32 weeks has increased the serum levels of 25(OH)D [29].

Vitamin D receptors (VDRs) are expressed in skeletal muscle, while vitamin D regulates gene expression and modulates the absorption of 25(OH)D by binding to its receptors [30]. Muscles also function as a storage area for vitamin D and maintenance of 25(OH)D levels as long as the body lacks exposure to sunlight [31]. Vitamin D maintains lung function and has an important role in cellular immunity [32]. Vitamin D deficiency can increase IgE in children aged 2–13 years, which is associated with the occurrence of inflammatory asthma [33]. The expression of the vitamin D receptor (VDR) gene, which encodes the enzyme vitamin D 1α-hydroxylase (CYP27B1) in respiratory epithelial cells, is important in preventing acute wheezing [34]. A vitamin D dose of 800 IU/day for 2 months in children with asthma can reduce the frequency and severity of asthma [35].

The long-term use of vitamin D may cause some adverse events (summarized in Table 2) [36]. In a clinical study of adverse-event vitamin D, including eosinophilia and neutropenia, the group that received intervention vitamin D3 4000 IU/day and the placebo group both revealed the same side effects [37]. Vitamin D functions to maintain eosinophil homeostasis [38]. Vitamin D deficiency causes allergies, where T-helper cells type 2 (Th2) will secrete interleukins, excessive production of immunoglobulin E (IgE), and eosinophil activation [39]. Vitamin D deficiency causes neutropenia [40]. Fluticasone, a corticosteroid, is a medication used to manage and treat asthma. Administration of corticosteroids may alter the vitamin D metabolism to 25(OH)D on calcium absorption [41,42]. Decreased vitamin D may stimulate a recurrence of diseases associated with the respiratory tract [42].

Vitamin D functions as a regulator of the innate and adaptive immune responses in the pathogenesis of autoimmune conditions, one of which is inflammatory bowel disease (IBD) [43]. In clinical studies, treatment with vitamin D for 4 weeks can reduce symptoms and improve the QoL of IBD patients [44]. Vitamin D can cause gastrointestinal side effects, including nausea, increased thirst, dry mouth, loss of appetite, and constipation [3]. Patients with symptoms of VDT will experience vomiting, dehydration, pain, and loss of appetite. These symptoms have recently been reported in patients with serum vitamin D concentrations ranging between 150 and 1220 ng/mL [16]. Vitamin D also causes side effects such as drowsiness, headaches, and unusual feelings of tiredness [3].

1,25(OH)_2_D (1,25-dihydroxy vitamin D, calcitriol), an active metabolite of vitamin D, can increase calcium absorption and increase calcinuria [45]. Hypercalcemia occurs in up to 4% of the population who consume excess calcium and/or vitamin D [46]. Hypercalciuria is a metabolic disorder in children characterized by the formation of calcium oxalate stones [47]. Excessive intake of vitamin D causes hypercalcemia and hypercalciuria due to the formation of 25(OH)D which binds to VDRs [46]. Mild hypercalcemia is characterized by symptoms of fatigue and constipation, whereas severe hypercalcemia causes nausea, vomiting, dehydration, confusion, and coma [48]. Hypercalcemia in children may present with hypotonia, lethargy, polyuria, dehydration, failure to thrive, seizures, and, in severe cases, kidney injury, pancreatitis, and unconsciousness [49].

**Table 2:** Adverse events of vitamin D in pediatrics.

Total Number of Patients	Age (Years)	Clinical Feature	Treatment	Duration (Weeks)	Adverse Events (AEs)	Refs.
192 (only 180 completed the study)	6–16 (mean age 9.8)	Persistent asthma and low vitamin D levels	Vitamin D3 4000 IU/day (n = 96) or placebo (n = 96) and maintained with fluticasone propionate: 176 μg/day (for age 6–11 years) or 220 μg/day (for age 12–16 years)	48	There were 36 participants (37.5%) in the vitamin D3 group and 33 (34.4%) in the placebo group who had 1 or more severe exacerbation. Serious AEs included hospitalizations (9 in each group), eosinophilia (1), and severe neutropenia (1).	[37]
63 (only 48 completed the study, and 1 withdrew for AEs)	8–18 (mean age 14.8)	IBD and baseline 25(OH)D ≥ 20 ng/mL	Arm A received 400 IU of oral vitamin D2/day (n = 32). Arm B received 1000 IU/day in the summer/fall and 2000 IU/day in the winter/spring (n = 31) Participants of arm B were notified via a phone call to change their vitamin D dose on the date such change was due. All participants received daily calcium supplementation: 800 mg elemental calcium (for age < than 11 years) and 1200 mg (for age ≥ 11 years).	24	Minor AEs (drowsiness, nausea and vomiting, dryness of mouth, increased thirst, persistent headache, constipation, loss of appetite, increased frequency of urination, bone and muscle pain, etc.) in both groups: 19 (59%) in arm A and 15 (48%) in arm B. More participants in arm A developed a C-reactive protein level of > 1 mg/dL and IL-6 > 3 pg/mL.	[3]
865 (patients at high risk of vitamin D deficiency and children with rickets were excluded)	1–5	Asthma based on clinical signs of airflow obstruction and reversibility according to Canadian guidelines; a recent history of asthma exacerbations requiring OCS (≥1 in the past 6 months or ≥2 in the past year, documented in pharmacy and/or medical records); frequent URTIs (≥4 in the past year) and URTIs identified by parents as the main asthma trigger	Intervention group participants received a 2 mL oral bolus of 100,000 IU vitamin D3 (50,000 IU cholecalciferol/mL) at randomization in the fall or early winter, followed by a second 2 mL oral bolus of 100,000 IU vitamin D3 3.5 ± 0.5 months later. Participants also receive a total of five 50 mL coded bottles, containing a 400 IU vitamin D3/mL preparation, to be administered at a dose of 1 mL/day using a dropper, for 7 ± 0.5 months. Each bottle contains 50 daily doses. Placebo group participants received an identical 2 mL placebo bolus and daily dose of 1 mL placebo preparation, with administration timing identical to the intervention group.	28	Hypercalciuria and hypercalcemia	[50]

AE: adverse event; IBD: inflammatory bowel disease; OCS: oral corticosteroids; URTIs: upper respiratory tract infections.

## 3. Pharmacodynamics

In the body, vitamin D undergoes a two-stage conversion into its active forms. One of them. vitamin D3 (cholecalciferol), is delivered in the bloodstream to attach to the vitamin D-binding protein. Within hours after its intake from meals, it is activated mainly via the liver to 25(OH)D, and then the kidney to 1,25(OH)_2_D (calcitriol). Generally, it is oxidized by CYP24A1 to become a more polar compound and is eliminated. A randomized open-label study of pharmacodynamics of vitamin D in healthy participants with deficiency demonstrated an elevation of 1,25(OH)_2_D levels at day 28, day 53, day 84, and day 112 compared to baseline; however, the 24,25(OH)_2_D was found unchanged over time [51].

Vitamin D stimulates the synthesis of FGF23, calbindin-D9K, calbindin-D28K, and Ca^2+^-ATPase in the serosa plasma membrane. Calbindin-D9K promotes Ca^2+^ extrusion by Ca^2+^-ATPase; the precise role of calbindin-D28K is unknown. Vitamin D’s major function is to boost intestinal Ca^2+^ absorption, which indirectly improves bone mineralization. As a result, PTH and vitamin D work separately to promote bone resorption [51]. Nuclear receptor VDR is expressed by osteoblasts, which are responsible for bone formation, and vitamin D stimulates the production of numerous osteoblast proteins, including osteocalcin, a vitamin K-dependent protein with -carboxyglutamic acid residues, and IL-1, a lymphokine that promotes bone resorption [52,53]. As a result, the current consensus is that vitamin D is a bone-mobilizing hormone rather than a bone-forming hormone. In a healthy situation, osteoblast and osteoclast activities are intertwined. Osteoporosis is a disorder in which this coupling is disrupted; osteoblast responsiveness to vitamin D is severely weakened, osteoclast activity predominates, and bone resorption outnumbers bone creation [52].

## 4. Pharmacokinetics

### 4.1. Absorption

The gastrointestinal system absorbs vitamin D compounds well with the aid of bile acid. Vitamin D in the forms of both ergocalciferol and cholecalciferol are equally absorbed, whereas 25(OH)D is better absorbed than cholecalciferol and ergocalciferol. It should be noted that the administration of vitamin D along with sucrose polyesters (Olestra) and/or tetrahydrolipstatin (Orlistat, an anti-obesity drug) may reduce the absorption of vitamin D [54].

Furthermore, vitamin D levels may decrease in patients with poor fat absorption. SR-BI (Scavenger Receptor Class B Type 1), CD36 (Cluster Determinant 36), and NPC1L1 (Niemann-Pick C1-Like 1) are proteins involved in vitamin D absorption. They are also implicated in apical uptake of cholesterol and other lipidic micronutrients, vitamin E, and carotenoids [54]. The incidence of vitamin D deficiency is also found in pediatric patients with human immunodeficiency virus (36%) due to the drug used, e.g., nucleoside reverse transcriptase inhibitors and protease inhibitors [55]. Malabsorption and inadequate nutrition also cause vitamin D deficiency which occurs as hypocalcemia in infancy, because the calcium demand of the body is not fulfilled, whereas in children it may lead to rickets and/or seizures or tetany [56].

The primary role of 1,25(OH)_2_D_3_ and VDR is the absorption of calcium in the intestine. However, the presumed mechanisms in this role still need further studies [53]. Therefore, an optimum amount of vitamin D is essential to maintain the absorption of calcium, which is normally 30–40%. Without sufficient levels of vitamin D, the body will only consume 10–15% of exogenous calcium [57]. A reduction of active calcium absorption occurs when the level of 25(OH)D is less than 20 nmol/L. A high calcium intake can elevate the half-life of 25(OH)D [58].

Moreover, the consequences of calcium deficiency (marked by urinary calcium/creatinine <0.2 mmol/mmol) are similar to those of vitamin D deficiency. It is linked with secondary increased 1,25(OH)2D levels, which reduces bone formation and redirects calcium towards the serum [59].

### 4.2. Distribution

High-affinity vitamin D-binding protein (DBP) is responsible for 85% of vitamin D metabolite transport, while low-affinity albumin accounts for the remaining 15% [60]. Even while 25(OH)D and 1,25(OH)_2_D have the highest affinity for their binding to DBP, all vitamin D metabolites can utilize this binding site [57]. According to the free hormone theory [61,62], researchers have long assumed that free 25(OH)D (representing 0.03% of the metabolites) and 1,25(OH)_2_D (representing 0.4% of the metabolites) are the sole active hormones entering cells. This hypothesis was based on a study of knockout mice for Lrp2 (encoding for megalin) with very bad osteomalacia and poor survival, revealing the important function of the DBP binding capacity in the kidney [63]. However, the free hormone concept has been updated lately, at least in some organs such as the kidney. Megalin, a large transmembrane protein, has been demonstrated to be localized to the apical side of proximal tubule cells, where it functions as a receptor for the complex vitamin D-DBP, alongside tubulin and disabled-2 proteins [64].

### 4.3. Metabolism

Vitamin D has two separate hydroxylation stages to obtain 1,25(OH)_2_D. The initial hydroxylation, at the C_25_ position, is a substrate-dependent and unregulated process that predominantly, but not solely, takes place in the liver. In fact, many enzymes exhibit 25-hydroxylase activity, but CYP2R1 has been identified as playing the largest role in the liver. Hepatocytes have CYP2R1 in their microsomal P450 fraction [65,66]. The CYP2R1 gene encodes a 501-amino-acid protein and is found on chromosome 11p15.2 (15.5 kb) in humans. Approximately half of the normal 25(OH)D levels are maintained by other 25-hydroxylase enzymes in CYP2R1 knockout mice [67].

Hydroxylation by CYP2R1 in the liver produces 25-OH-cholecalciferol (25(OH)D_3_) and 25-OH-ergocalciferol (25(OH)D_2_). The primary circulating forms of vitamin D3 are 25(OH)D_3_ (biological t_1/2_ = 19 days and normal steady-state values of 15–50 ng/mL) and 25(OH)D_2_ (biological t_1/2_ =13 days). 25(OH)D enters the circulation after being produced in the liver and is transported by vitamin D-binding globulin. Final activation occurs in the proximal tubules of the kidney, in which the enzyme 25-hydroxyvitamin D-1-hydroxylase (CYP27B1) works to convert 25(OH)D_3_ to 1,25(OH)_2_D [50]. The kidney was formerly assumed to be the sole source of 1,25(OH)_2_D due to the discovery of renal CYP27B1 [68]. In response to calcium and phosphate concentrations, the renal form of CYP27B1 is regulated by at least three hormones, with a critical role in calcium–bone metabolism. PTH increases hydroxylation, while 1,25(OH)_2_D inhibits it [69,70]. Clearly, a lack of vitamin D, calcium, or phosphate in the diet increases the 1-hydroxylation of 25(OH)D_3_, resulting in the synthesis of physiologically active 1,25(OH)_2_D_3_. In contrast, when Ca^2+^ concentrations rise, 24-hydroxylation inactivates 25(OH)D_3_. Similar responses occur in the presence of 25(OH)D_2_ [52]. 1,25(OH)_2_D has terminal half-lives that range from 5–10 h in healthy people to 15–30 h in dialysis patients [71].

In the previous few decades, researchers have described more than 50 different vitamin D metabolites, some of which have shown to be of particular interest due to their biological activity. CYP24A1, encoded by the CYP24A1 gene on chromosome 20q13.2, is the most well-known catabolic enzyme [69]. 24R,25(OH)_2_D and 1,24,25(OH)_3_D are the products of CYP24A1’s hydroxylation of 25(OH)D and 1,25(OH)_2_D, respectively. These compounds undergo sequential hydroxylation catalyzed by the same enzyme to generate a succession of 24- and 23-hydroxylated analogues. The inert calcitroic acid, or 26,23-lactone, is then passed out of the body via bile or urine. 1,25(OH)_2_D and FGF23 stimulate CYP24A1, while parathyroid hormone and low calcium levels suppress it. CYP24A1 is essential for the local regulation of vitamin D activity and has been found in various tissues expressing VDR [72].

Other, less significant metabolic pathways have been described and need to be evaluated in addition to CYP24A1. Recent research has uncovered the C-3 epimerization process, which results in the formation of several C-3 epimer metabolites in which the C-3 hydroxyl group is oriented alpha rather than beta. High expression of epimeric metabolites has been observed, especially in newborns and young children; nevertheless, the physiological relevance of this redundant pathway remains unclear [73].

High-affinity vitamin D-binding protein (DBP) is responsible for 85% of vitamin D metabolite transport, while low-affinity albumin accounts for the remaining 15% [60]. Even while 25(OH)D and 1,25(OH)_2_D have the highest affinity for binding DBP, all vitamin D metabolites can utilize this binding site [61]. According to the free hormone theory [62], researchers have long assumed that free 25(OH)D (representing 0.03% of the metabolites) and 1,25(OH)_2_D (representing 0.4% of the metabolites) are the sole active hormones entering cells. However, the free hormone concept has been updated lately, at least in some organs such as the kidney. Megalin, a large transmembrane protein, has been demonstrated to be localized to the apical side of proximal tubule cells, where it functions as a receptor for the complex vitamin D-DBP, alongside tubulin and disabled-2 proteins [64,65].

### 4.4. Elimination

By hydroxylating 25(OH)D and 1,25(OH)_2_D, CYP24A1 generates 24R,25(OH)_2_D and 1,24,25(OH)_3_D. Sequential hydroxylation of these substances by the same enzyme results in a series of 24- and 23-hydroxylated. Calcitroic acid or 26,23-lactone, is a chemically inert compound that is excreted from the body. Most of the vitamin D in the form of vitamin D3 is eliminated via the bile into the feces. Only a small amount is excreted via urine and the excretion products have not been identified, thus need further studies [72,73].

## 5. Toxicokinetics and Case Reports of Vitamin D Toxicity in Pediatrics

Toxicokinetics describes the same principle as pharmacokinetics, but the mechanism is applied specifically to toxicants or when the kinetic data of the therapeutic agent are at a dose that allows toxicity to occur [74]. Toxicokinetics is also associated with the ratio of external exposure concentration to biologically target site-effective dose concentration [75,76]. Toxicokinetics studies are also usually carried out to measure concentration, monitor biotransformation, and trace the formation of metabolites that may cause adverse effects in order to assess the potential toxic effects of a drug [77]. Toxicokinetics is the body’s tolerance limit for receiving the effects of a compound and its metabolites so that it has a negative effect on the body [76]. An example is the overdose phenomenon, where a high level of drug intake significantly elevates drug concentration in the blood and tissues [78].

VDT is often associated with overdosage because of the over-fortification of milk and food products [15,79] or high-dose supplements intake in pediatrics. Some supplements containing vitamin D exists as unregulated or unlicensed formulations [80]. Supported by the lack of public education about permissible limits of vitamin D intake, many cases in VDT have been reported [15]. VDT can be caused by a high dose of vitamin D combined with calcium which will promote hypercalcemia [81]. The symptoms of hypercalcemia are neurological (confusion, irritability, restlessness, and apathy, and in severe cases, can cause psychosis, stupor, and coma); cardiovascular (hypertension, shortened QT interval which indicates arrythmia, elevated ST segment, bradyarrhythmia, and first-degree heart block), gastrointestinal (abdominal pain, nausea, ulcer, vomiting, polydipsia, anorexia, constipation, and pancreatitis), and kidney symptoms (hypercalciuria, acute kidney injury (AKI), polyuria, polydipsia, dehydration, and nephrocalcinosis) [82,83]. Weakness, anorexia, loss of skin turgor, weariness, bone aches and dry mucous membranes (due to dehydration), changes in mental status, stomach soreness without rebound, rigidity, or guarding may occur as non-specific symptoms [83]. Several VDT cases in pediatrics are presented in Table 3.

Management of VDT which was applied in some cases is tabulated in Table 3, including the discontinuation of vitamin D intake, use of activated charcoal, fluid therapy, furosemide, prednisone, calcitonin therapy, IV hydration with normal saline, and dextrose solution. Activated charcoal is a natural adsorbent which can make a tight binding with a lipophilic compound such as a vitamin [88]. The adsorbent can also be used as orogastric lavage and gastrointestinal decontamination in hypercalcemia patients [89]. Furthermore, VDT may be overcome by the calciuresis method, which increases the glomerular filtration rate and inhibits the reabsorption of calcium proximal nephron [90,91,92,93]. The therapy was carried out by administering the loop diuretics and corticosteroids and using physiologic saline. Loop diuretics such as furosemide play a role in reducing calcium reabsorption in the loop of Henle [94,95]. In addition, glucocorticosteroids are used to diminish the reabsorption of calcium to elevate the excretion process [88]. Another therapy, calcitonin and bisphosphonate, are drugs which be used in anti-resorptive therapy. In particular, the drugs can be applied to treat moderate hypercalcemia cases. The drugs can elevate osteoclastic bone resorption as a result of 1,25(OH)_2_D activity in bone tissue. Calcitonin produces a faster effect than bisphosphonates, but bisphosphonates provide a longer therapeutic effect. Some reports have shown that bisphosphonates intake is more effective in overcoming VDT than calcitonin, especially in pediatrics [82].

## 6. Toxicodynamics

Hypercalcemia is a disordering homeostatic calcium disease with high levels of free 1,25(OH)_2_D in the blood and decreased expression of PTH. Generally, vitamin D has two forms: Vitamin D2 (ergocalciferol) and D3 (cholecalciferol) [96]. Vitamin D2 is produced from food intake and supplements, while vitamin D3 is mostly obtained from the conversion of 7-dehydrocholesterol (7-DHC) in UVB exposure. Vitamin D acquired from food intake is integrated into chylomicron and binds the vitamin D-binding protein (VDBP) to circulate into vena blood vessel. The vitamin D will then be transported into the liver and adipose tissue. In the liver, vitamin D will be metabolized by 25-hydroxylase to obtain the 25(OH)D form as vitamin D inactive form [97,98].

Commonly, the levels of 25(OH)D in the blood are analyzed as a standard for determining vitamin D status in the human body. 25(OH)D circulates to the kidneys and will be activated by 1-α-hydroxylase (1-OHase) to produce 1,25(OH)_2_D, which is released into the circulation as a free form. VDR binds 1,25(OH)_2_D to stimulate the absorptions of calcium and phosphor in the intestine. The elevation of serum calcium level can be detected by CaSR (calcium-sensing receptor) to facilitate negative feedback in the parathyroid gland and diminish PTH secretion. In normal situations, negative feedback of the parathyroid is an essential role to maintain the homeostasis of calcium absorption, but a very low concentration of PTH is a hypercalcemia symptom. PTH is the primary calciotropic hormone and acts to elevate calcium levels by promoting osteoclastic activity in bones, raising the reabsorption of calcium in distal tubules, and collecting ducts of kidney. In addition, 1,25(OH)_2_D also plays a role in increasing the expression of D-24-hydroxylase (24-OHase) to prevent 24-OHase excretion in bile and causes mature osteoclast induction in the osteoblast through the expression of nuclear ligand factor-κB receptor activator (RANKL) [49,96].

**Figure 1:**
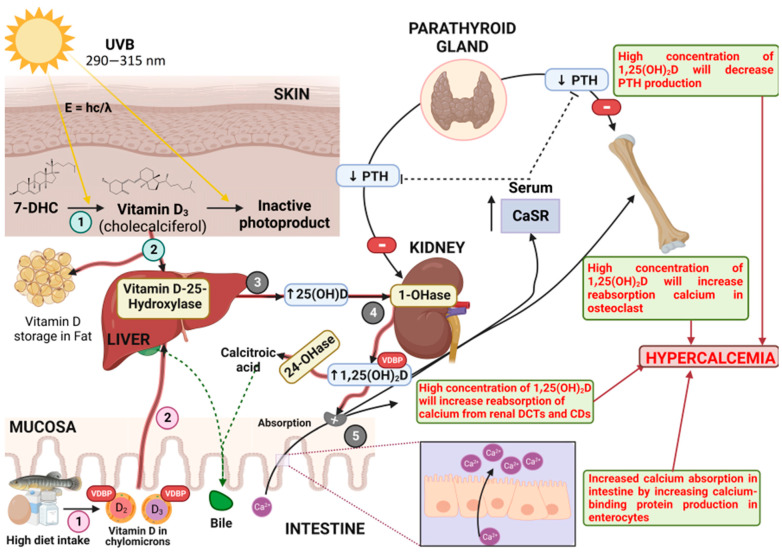
Toxicodynamic of Vitamin D in High-Dose Intake: (1) Vitamin D from dietary meals and from the conversion of 7-DHC in the skin by UVB exposure is converted into vitamin D_2_ and integrated into chylomicrons; (2) vitamin D in chylomicrons is transported to the liver or stored in adipose tissue; (3) the liver enzyme converts vitamin D_2_ and vitamin D_3_ to 25(OH)D, which is distributed in the blood; (4) it is then brought to the kidney, which is eventually activated to produce 1,25(OH)D; (5) finally, it is released into the circulation as a free form and binds to VDR.

High levels of 25(OH)D in the circulation will promote the activation of VDR, which can replace 1,25(OH)_2_D in the binding protein to produce the free form of 1,25(OH)_2_D in high concentration. Vitamin D and metabolite derivatives at a moderate level can trigger tissue elimination causing hypercalcemia which is marked by persistence [86,98]. The elevation of osteoclastic bone resorption, renal calcium tubules [90,91,93], intestinal calcium intake (through increased production of calcium-binding protein in enterocytes), and decreased PTH synthesis are the regular mechanisms of hypercalcemia [99]. The toxicodynamic of vitamin D is illustrated in Figure 1.

However, there may be a limitation in measuring blood 25(OH)D levels; such a major problem is due to the physicochemical property of the molecule. It is the most hydrophobic compound due to the fact that it exists in two forms, 25(OH)D_2_ and 25(OH)D_3_ [100].

## 7. Vitamin D in Pregnancy

The incidence of vitamin D deficiency during pregnancy has increased. It was reported that there is an association between low vitamin D levels and adverse maternal outcomes, e.g., pregnancy-induced hypertension, gestational diabetes mellitus, recurrent pregnancy loss, preterm birth, primary Caesarian section, and postpartum depression. Randomized controlled trials on vitamin D supplementation recommended a safe dose of 2000 to 4000 IU/day for pregnant women [101,102]. Furthermore, by employing mathematical calculation, it was decided that circulating vitamin D levels should be higher than 40 ng/mL during pregnancy [103]. Serum 25(OH)D rapidly decreases during pregnancy due to the physiological needs of the fetus. Vitamin D supplementation in pregnant women with low vitamin D status may help the growth of the fetus and diminish the risks of small-for-gestational-age, preeclampsia, preterm birth, and gestational diabetes [101].

## 8. Conclusions

The dose and duration of vitamin D usage in pediatrics need to be considered because the child’s digestive system is developing. It is suggested to monitor the infant’s nutritional vitamin D status, particularly when maternal vitamin D deficiency occurs, by measuring both 25(OH)D and 1,25(OH)D_2_ because these molecules are the most abundant metabolites in circulation. Vitamin D supplements given in the long term may lead to VDT, with symptoms such as nausea, increased thirst, and constipation. VDT also causes neutropenia, eosinophilia, hypercalciuria, and hypercalcemia. VDT in pediatrics can be managed by discontinuing vitamin D intake; using activated charcoal, furosemide, prednisone, and calcitonin; rehydration using intravenous sodium chloride 0.9%; and dextrose fluid therapy.

## Figures and Tables

**Table 1:** Recommended dose of vitamin D supplementation for pediatrics [20,21].

Pediatrics	Dose of Vitamin D Supplementation
Age 0 to 12 months breastfed or partially breastfed babies	A dose of 400 IU/day starting from the first few days.
	Supplementation should be continued unless the infant is weaned to at least 1000 mL of vitamin D-fortified formula or whole milk/day.
	All infants ingesting less than 1000 mL of formula milk/day or non-breastfed infants should receive a vitamin D supplement of 400 IU/day.
Age 1 year to 18 years	A dose of 600 IU/day in the absence of sun exposure.
	Older children who are ingesting less than 1000 mL of vitamin D-fortified formula or whole milk/day should receive a vitamin D supplement of 400–600 IU/day.

**Table 3:** Case reports of VDT in pediatrics.

Information about the Patients (Age in Months)	Results of Examination	Toxicity Management	Refs.
A total of 15 pediatric patients aged 24–60 months (mean age 46.53 ± 10.14 months) with a history of ingestion of more than 1500 IU/day of vitamin D supplements	The mean ingested dose was 8.13 ± 4.54 soft gelatin capsules or 406,700.7 ± 227,400.1 IU vitamin D. One patient had ingested 500,000 IU vitamin D with serum Ca level of 12.5 mg/dL. Eight (53.3%) cases had 25(OH)D levels > 100 ng/mL. The mean serum 25(OH)D levels of the patients was higher than normal: 111.3 ± 113.6 ng/mL (normal 30–100 ng/mL). There was no significant difference between variables in patients with and without a high level of 25(OH)D.	There were 8 patients (53.3%) who were hospitalized and treated with activated charcoal and fluid therapy, discontinued consumption of vitamin D supplements, kept low-calcium and vitamin D diet, took more liquid for at least one month, and were monitored for 25(OH)D levels.	[84]
Two infants with an identical presentation. One was a 3.5-month-old Caucasian female and the other a 2.5-month-old Caucasian male. The patients were exclusively breastfed and had received OTC vitamin D supplementation higher than the recommended dose. The female infant was given vitamin D3 2000 IU/day for 2.5 months, while the male infant was given vitamin D3 20,000 IU/day for 1.5 weeks	Physical examination of both patients showed evidence of moderate dehydration. Laboratory analysis of the female infant: Serum Ca: 21 mg/dL (normal 8.8–11.2 mg/dL) 25(OH)D: 644 ng/mL (normal 30–100 ng/mL) PTH: <1 pg/mL (normal 14–72 pg/mL) Laboratory analysis of the male infant: Serum Ca: 15 mg/dL (normal 8.5–10.1 mg/dL) 25(OH)D: 680 ng/mL (normal 30–100 ng/mL) 1,25(OH)_2_D: 166 pg/mL (normal 15–75 pg/mL) PTH: <7 pg/mL (normal 15–65 pg/mL)	The infants received IV hydration with normal saline and dextrose-containing solution at the PICU where they received furosemide 1 mg/kg/dose every 8 h and prednisone 1 mg/kg/day. The male infant received calcitonin 4 IU/kg × 1 dose. They both exhibited improvement of hypercalcemia after 2 to 3 days of treatment. On discharge, the serum Ca of the female infant was 11 mg/dL and the male infant was 10.8 mg/dL; both were clinically improved.	[85]
A 3-month-old Asian–American male infant who had been exclusively breastfed. The oral vitamin D supplementation was started on day 5 (400 IU/day), but the parents had made a mistake when administering a new brand of infant vitamin D liquid preparation to a 30-fold overdose of vitamin D (12,000 IU) daily for 20 days	The infant had no history of irritability, constipation, or abnormal movements. Laboratory analysis: Serum Ca: 10.5 mg/dL (normal 8.5–10.1 mg/dL) Phosphorus: 6.4 mg/dL (normal 4.5–6.5 mg/dL) Electrolyte panel, creatinine, and blood urea nitrogen were normal. Serum 25(OH)D: 422 ng/mL (normal 30–100 ng/mL) Serum 1,25(OH)_2_D: 61 pg/mL (normal 27–71 pg/mL) PTH: <3 pg/mL (normal 15–65 pg/mL)	The parents were asked to stop giving vitamin D to the infants.	[86]
A 3-month-old male infant with severe anorexia, vomiting, and weight loss. The infant was born at term, weighing 2.3 kg, and had been exclusively breastfed with no medical problems. The infant was exposed to 40,000–50,000 IU of vitamin D supplement/day, which represents 50-fold the Upper tolerable Level (UL) recommended.	At the time of admission, the infant weighed 4.5 kg with no fever. HR 109 beats/minute, BP 107/85 mmHg, oxygen saturation 99%. Laboratory analysis: Serum Na: 139 mmol/L Serum K: 4.4 mmol/L Alkaline reserve: 21 mmol/L Hb: 9.1 g/dL Leukocytes: 11.11 g/L Platelets: 471 g/L C-reactive protein: 13 mg/L with a negative procalcitonin Serum Ca: 3.08 mmol/L (normal 2.15–2.55 mmol/L) Serum albumin: 41 g/L (normal 34–42 mg/L) PTH: <18 ng/L (normal 18–88 ng/L) Serum 25(OH)D: > 400 ng/mL (normal 30–400 ng/mL) Serum 1,25(OH)_2_D: 200 pg/mL (normal < 182 pg/mL) Phosphate: 1.8 mmol/L (normal 1.6–2.4 mmol/L) Serum creatinine: 23 μmol/L (normal 15–37 μmol/L) Urea: 2.3 mmol/L (normal 1.8–6.4 mmol/L) Blood gases analysis and liver function tests were within normal range.	The patient was hospitalized, given IV hydration, and examined for immunoglobulin E (IgE) antibodies to cow milk, cranial ultrasonography, brain magnetic resonance imaging, and abdominal ultrasound. Because of a suspicion of urinary infection, IV antibiotic therapy was also given for 3 days. The patient was discharged after 2 weeks with a weight of 4.8 kg and a close monitoring of his serum Ca.	[87]

BP: blood pressure; HR: heart rate; IV: intravenous; OTC: over the counter; PICU: pediatric intensive care unit; PTH: parathyroid hormone. All values are for infants.

## Data Availability

All data generated and analyzed are included within this review article.
